# Mucoepidermoid carcinoma of the lung: a case report

**DOI:** 10.1186/1749-8090-6-132

**Published:** 2011-10-11

**Authors:** Masahiro Kitada, Yoshinari Matsuda, Kazuhiro Sato, Satoshi Hayashi, Kei Ishibashi, Naoyuki Miyokawa, Tadahiro Sasajima

**Affiliations:** 1 Department of Surgery, Asahikawa Medical University, Asahikawa, Japan; 2 Department of Clinical Pathology, Asahikawa Medical University, Asahikawa, Japan

## Abstract

Mucoepidermoid carcinoma of the lung (MEC) is a tumor of low malignant potential of bronchial gland origin. MEC and adenoid cystic carcinoma are both considered to be salivary gland-type neoplasms. MECs are comparatively rare with an incidence of all lung cancers. We recently encountered a case of this type of lung cancer. A 60-year-old man was found to have an abnormal shadow in the left lower lung field on a regular check-up for lung cancer at his company. Chest radiography and CT revealed a mass shadow measuring 30 mm in diameter in the left lower lung field. Bronchoscopy revealed a protuberant tumor in the S9 bronchus, leading to a diagnosis of low-grade MEC by transbronchial lung biopsy. He underwent left lower lobe resection and mediastinal lymph node dissection using VATS. Tumor cells had a scattering of mucus-producing epithelial components in papillary growth of stratified squamous epithelia with anisokaryosis and minimal pleomorphism, indicating a diagnosis of MEC. Because the postoperative course was good and the tumor was low-grade, no adjuvant treatment was administered. The patient has had no signs of tumor recurrence for 9 months, to date, since resection of the tumor

## Introduction

Mucoepidermoid carcinoma of the lung (MEC) is a tumor of low malignant potential of bronchial gland origin. MEC and adenoid cystic carcinoma are both considered to be salivary gland-type neoplasms. MECs are comparatively rare with an incidence of 0.1%-0.2% of all lung cancers, occurring mostly in young persons. MECs proliferate in a polyp-like form in the central bronchial lumen up to the segmental bronchus level. We report a case of MEC.

## Case

**Figure 1:**
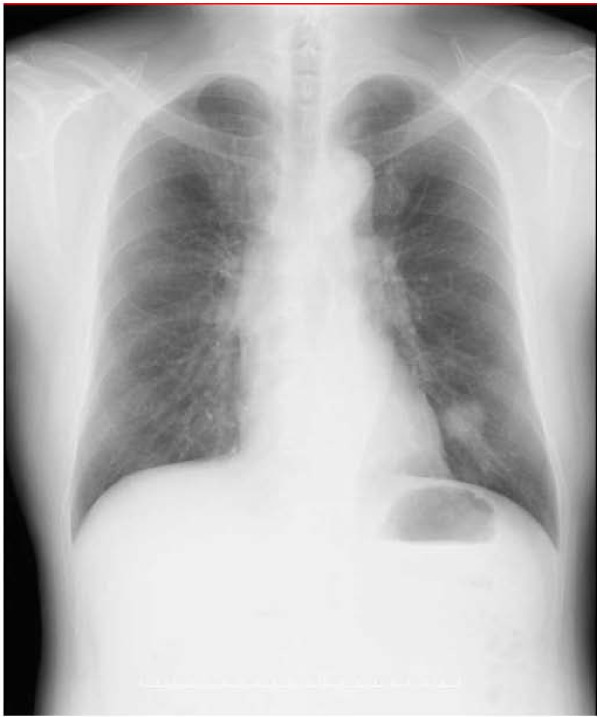
**Chest radiography revealed a mass shadow in the left lower lung field**.

**Figure 2:**
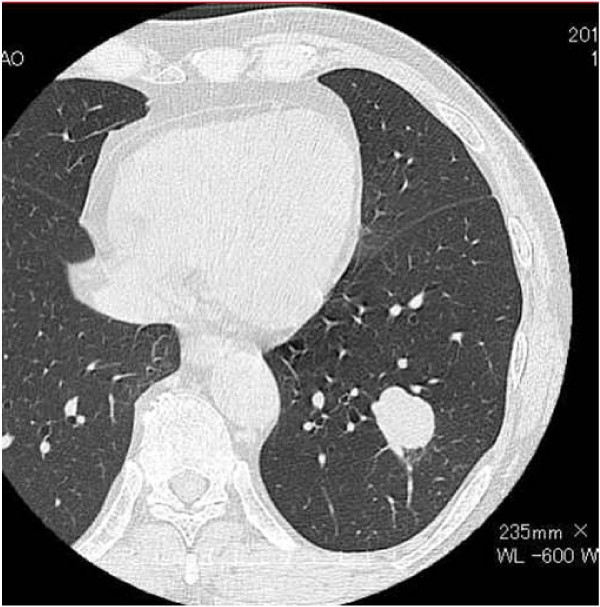
**CT showed a mass shadow measuring 30 mm in diameter in S9**.

**Figure 3:**
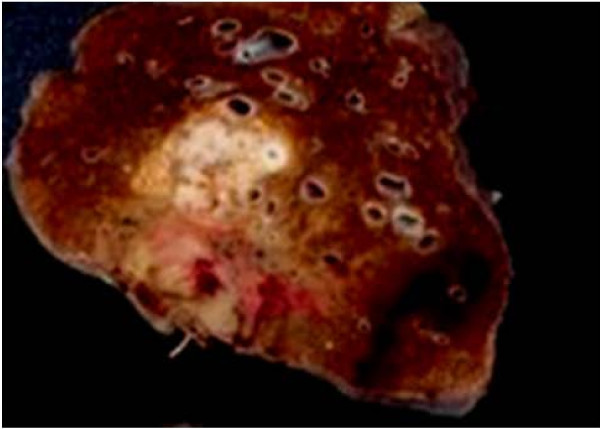
**The macroscopic specimen showed a mass localized into the arborization of the S9 bronchus and obstructive pneumonitis was accompanied at the peripheral lung**.

**Figure 4:**
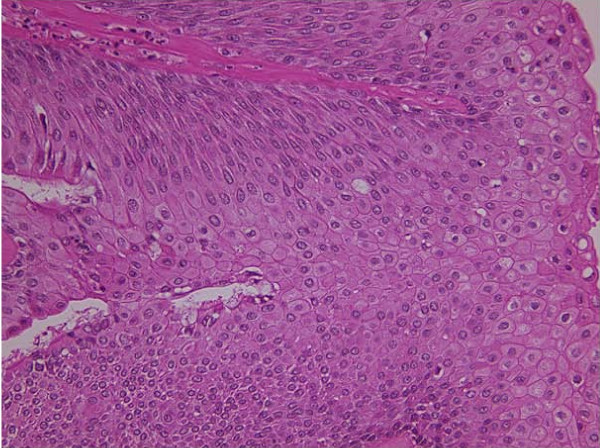
**Histological findings showing a mass diagnosed MEC**. (HE × 200).

**Figure 5:**
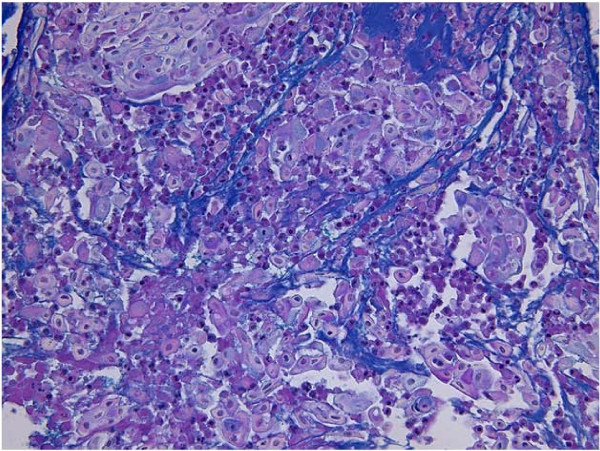
**Immunohistochemical examination revealed that tumor cells were positive for Periodic acid-Schiff stain (PAS) (×100)**.

A 60-year-old man was found to have an abnormal shadow in the left lower lung field on a regular check-up for lung cancer at his company. Chest radiography revealed a mass shadow measuring 30 mm in diameter in the left lower lung field (Figure 1), and chest CT showed a lobulated mass shadow measuring 30 mm in diameter in S9 (Figure 2). No mediastinal lymph node metastasis or other organ metastases were observed. His past and family histories were unremarkable. The patient had a smoking habit with a Brinkman index of 800. Blood tests showed no abnormal tumor markers. Bronchoscopy revealed a protuberant tumor in the S9 bronchus, leading to a diagnosis of low-grade MEC by transbronchial lung biopsy. The patient underwent left lower lobe resection and mediastinal lymph node dissection using VATS. The macroscopic specimen showed a mass localized into the arborization of the S9 bronchus and obstructive pneumonitis was accompanied the peripheral lung (Figure 3). Although histopathology disclosed most parts of the tumor to be localized within a branch of the B9 bronchus, some parts had invaded the pulmonary tissue. Because there were features of obstructive pneumonia in the S9 area, the actual tumor diameter was deemed to be 2.4 cm. Tumor cells had a scattering of mucus-producing epithelial components in papillary growth of stratified squamous epithelia with anisokaryosis and minimal pleomorphism, indicating a diagnosis of MEC (Figure 4). Immunohistochemical examination revealed that tumor cells were positive for Periodic acid-Schiff stain (PAS) (Figure 5). The mitotic count was about 1-2 per 10 HPP, and ki-67 expression was about 15%, consistent with a low-grade tumor. The tumor had a keratin profile of CK7 (+), CK20 (-), and CK5/6 (+), and was determined to be a primary lung cancer (Figure 5). Lymph-vascular invasion, vascular invasion, and lymph node metastasis were negative. Because the postoperative course was good and the tumor was low-grade, no adjuvant treatment was administered. The patient has had no signs of tumor recurrence for 9 months, to date, since resection of the tumor.

## Discussion

MEC is a malignant tumor of bronchial gland origin first described by Smetana in 1952 [1], with a presumed incidence of 0.1%-0.2% of all lung cancers [2]. This tumor has been reported to occur in relatively young persons as compared with most other lung cancers [3]. MEC generally occurs in the central bronchial region, and many of these tumors are detected based on symptoms such as coughing, sputum, bloody sputum and wheezing, and chest pain, chest oppression and fever associated with obstructive pneumonia. Because this disease originates from glandular tissue identical with salivary glands located in the submucosa of the trachea and bronchus, it is included among carcinomas of salivary-gland types along with adenoid cystic carcinoma according to the WHO histological classification of lung cancer. MEC is characterized by a mixture of mucus-producing, glandular and squamous epithelial cells, as well as intermediate cells with both properties at various percentages, and by various growth patterns such as cystic, papillary, and solid structures [4]. Mucus-producing cells form lumens in some cases. Most MEC cases show low-grade 1-2 nuclear atypia with many squamous epithelial components, while high-grade cases have predominantly mucus-producing cells. Therefore, MEC has been considered difficult to differentiate from adenosquamous cancer [5].

Radical surgery based on lung cancer treatment is performed for MEC, and in recent years this operation has frequently been performed using VATS [6]. In addition, if this tumor arises in the central bronchus, resection based on bronchoplasty considering preservation of pulmonary function is also conducted. Patients with low-grade MEC generally have a good prognosis, with a 5-year survival rate of 95%, and adjuvant treatment is considered unnecessary. However, effective treatment measures for high-grade tumors have not been established, and these cases reportedly have a poor prognosis [3,7,8]. Under such circumstances, there are several reports on the efficacy of the tyrosine-kinase inhibitor Gefitinib in patients with EGFR gene mutations [9,10], and this molecularly-targeted therapy is likely to improve prognoses of cases with progressive high-grade and recurrent MEC. Therefore, EGFR gene mutations appear to be important.

## Conclusion

MEC is a comparatively rare low-grade tumor, which reportedly carries a good prognosis. However, the possibility of high-grade MEC should be kept in mind.

## Consent statement

Informed consent was obtained from the patient for publication of this case report and accompanying images. A copy of the written consent is available for review by the Editor-in-Chief of this journal.

## Competing interests

The authors declare that they have no competing interests.

## Authors' contributions

MK have operated this case and analyzed all data. YM, KS, SH, KI did the assistant of the operation. NM diagnosed h the pathology of this case.

TS was the professor of the surgical science and had a guide.

All authors read and approved the final manuscript.
